# Correlates of knee bone marrow lesions in younger adults

**DOI:** 10.1186/s13075-016-0938-9

**Published:** 2016-01-26

**Authors:** Benny Antony, Alison Venn, Flavia Cicuttini, Lyn March, Leigh Blizzard, Terence Dwyer, Andrew Halliday, Marita Cross, Graeme Jones, Changhai Ding

**Affiliations:** Menzies Institute for Medical Research, University of Tasmania, 17 Liverpool Street, Hobart, TAS 7000 Australia; Department of Epidemiology and Preventive Medicine, Monash University, 99 Commercial Road, Melbourne, VIC 3004 Australia; Institute of Bone and Joint Research, University of Sydney, Sydney, NSW 2006 Australia; Murdoch Children’s Research Institute, 50 Flemington Road, Melbourne, VIC 3052 Australia; Department of Radiology, Royal Hobart Hospital, 48 Liverpool Street, Hobart, TAS 7000 Australia

**Keywords:** Young adults, Cholesterol, Physical activity, Bone marrow lesions, Cartilage defects, Meniscal lesions

## Abstract

**Background:**

Subchondral bone marrow lesions (BMLs) play a key role in the pathogenesis of osteoarthritis (OA) and are associated with pain and structural progression in knee OA. However, little is known about clinical significance and determinants of BMLs of the knee joint in younger adults. We aimed to describe the prevalence and environmental (physical activity), structural (cartilage defects, meniscal lesions) and clinical (pain, stiffness, physical dysfunction) correlates of BMLs in younger adults and to determine whether cholesterol levels measured 5 years prior were associated with current BMLs in young adults.

**Methods:**

Subjects broadly representative of the Australian young adult population (*n* = 328, aged 31–41 years, female 48.7 %) underwent T1- and proton density-weighted fat-suppressed magnetic resonance imaging (MRI) in their dominant knee. BMLs, cartilage defects, meniscal lesions and cartilage volume were measured. Knee pain was assessed by Western Ontario and McMaster Universities Osteoarthritis Index (WOMAC) and physical activity was measured by the International Physical Activity Questionnaire (IPAQ). Cholesterol levels including high-density lipoprotein (HDL) were assessed 5 years prior to MRI.

**Results:**

The overall prevalence of BML was 17 % (grade 1: 10.7 %, grade 2: 4.3 %, grade 3: 1.8 %). BML was positively associated with increasing age and previous knee injury but not body mass index. Moderate physical activity (prevalence ratio (PR):0.93, 95 % CI: 0.87, 0.99) and HDL cholesterol (PR:0.36, 95 % CI: 0.15, 0.87) were negatively associated with BML, while vigorous activity (PR:1.02, 95 % CI: 1.01, 1.03) was positively associated with medial tibiofemoral BMLs. BMLs were associated with more severe total WOMAC knee pain (>5 vs ≤5, PR:1.05, 95 % CI: 1.02, 1.09) and WOMAC dysfunction (PR:1.75, 95 % CI: 1.07, 2.89), total knee cartilage defects (PR:2.65, 95 % CI: 1.47, 4.80) and total meniscal lesion score (PR:1.92, 95 % CI: 1.13, 3.28).

**Conclusions:**

BMLs in young adults are associated with knee symptoms and knee structural lesions. Moderate physical activity and HDL cholesterol are beneficially associated with BMLs; in contrast, vigorous physical activity is weakly but positively associated with medial tibiofemoral BMLs.

**Electronic supplementary material:**

The online version of this article (doi:10.1186/s13075-016-0938-9) contains supplementary material, which is available to authorized users.

## Background

Osteoarthritis (OA) affects whole joint structure including subchondral bone that results in joint pain and dysfunction. Subchondral bone is rich in blood and nerve supply and there is emerging evidence to suggest that changes in bone precede cartilage damage, so that bone rather than cartilage may be the site initiating the significant pathophysiological events in OA [1, 2]. Subchondral bone marrow lesions (BMLs) play a key role in the pathogenesis of OA [3] and are associated with pain [4]. BMLs can regress and progress over time [5] and BML regression is associated with a decrease in knee pain [6, 7]. This makes BML an attractive target for treatment of OA [8] and it is being used in clinical trials as an outcome measure [9, 10]. BMLs in the tibiofemoral joint can predict both structural and clinical changes in knee OA [11, 12] and the natural history of BMLs has been explored in older adults [13]. Little is known about their determinants and clinical significance in younger population.

The prevalence of vascular disease is high among people with OA [14–16]. These diseases may share some common risk factors such as obesity, high low-density lipoprotein (LDL) levels and elevated total cholesterol [17]. Subchondral bone ischemia may be one of the mechanisms by which vascular pathology contributes to the development of BMLs [18]. Total cholesterol and triglycerides were associated with BMLs in women [19] and high-density lipoprotein (HDL) cholesterol seems to have a protective effect on incidence of BMLs in older adults [18]. Again, these studies are mostly performed in older adults and there are no studies in young adults.

The association of BMLs with physical activity is controversial. The current evidence, mostly in older adults, is mixed with some showing beneficial [20] and some detrimental effects [21, 22]; a recent systematic review found limited evidence overall for an association between physical activity and BMLs [23]. The associations between physical activity and BMLs in young adults have not been explored.

The aims of this study were, therefore, to describe the environmental (physical activity), structural (cartilage defects, meniscal lesions) and clinical (pain, stiffness, physical dysfunction) correlates of BMLs in younger adults and to determine whether cholesterol levels measured 5 years prior were associated with current BMLs in young adults.

## Methods

### Study population

Participants (*n* = 328, aged 31–41 years, female 48.7 %) were broadly representative of the Australian young adult population because they were originally part of the Australian Schools Health and Fitness Survey (ASHFS) of 1985. This survey was conducted in schools Australia-wide and the sampling procedures and methods of data collection are presented elsewhere [24]. The Childhood Determinants of Adult Health (CDAH) study is a 20-year follow-up study of the ASHFS conducted during 2004 to 2006 and included subjects of mean age 31 (*n* = 2410, age range 26–36 years) who underwent various measurements including their blood cholesterol levels. The current study, known as the CDAH Knee study was conducted during 2008–2010 and participants were originally involved with the CDAH study [25]. The CDAH participants (*n* = 764) living in Melbourne and Sydney were contacted and invited to participate in the CDAH Knee study. Eligibility criteria were assessed in subjects who agreed to participate (*n* = 529, response percentage 69 %). Exclusion criteria included the following: being pregnant; having diseases that may affect knee cartilage such as rheumatoid arthritis; having a contraindication for magnetic resonance imaging (MRI) including claustrophobia. Eighty subjects were excluded, and the remaining 449 subjects were asked to complete a short computer-assisted telephone interview with physical activity and knee pain questionnaires. Injury status to the knee was recorded in response to the question “Have you had a knee injury requiring non-weight-bearing treatment for more than 24 hours or surgery?” They were requested to have a MRI scan of their dominant knee at Epworth Hospital in Melbourne or North Shore Private Hospital in Sydney. Some participants (*n* = 119) did not undergo MRI after enrolling in the study due to the long distance that they would have needed to travel for MRI, work or family commitments, moving interstate, becoming pregnant by the time of MRI, or changing their mind. Two subjects’ MRIs were not readable and they were excluded. This study was approved by the Southern Tasmania Health and Medical Human Research Ethics Committee, the Monash University Human Research Ethics Committee and the Northern Sydney and Central Coast Area Human Research Ethics Committee, and all participants provided written informed consent.

### Anthropometric measurements

Weight was measured to the nearest 0.1 kg in CDAH study as well as in CDAH Knee study with shoes, socks, and bulky clothing removed. Height was measured to the nearest 0.1 cm (with shoes and socks removed) using a stadiometer. Waist circumference was measured to the nearest 0.1 cm. Body mass index (BMI) was calculated as kilograms of weight per square metre of height.

### Physical activity measurements

Physical activity status (at work, as part of house and yard work, to get from place to place, in spare time for recreation, exercise and/or sports) in the past 7 days was recorded by requesting the participants in the CDAH Knee study to complete a short version of the International Physical Activity Questionnaire (IPAQ). Physical activities were calculated to represent minutes per week of vigorous physical activity, moderate physical activity, walking and total physical activity. Vigorous physical activity was recorded using the question: “think about all the vigorous activities which take hard physical effort that you did in the last 7 days. Vigorous activities make you breathe much harder than normal and may include heavy lifting, digging, aerobics, or fast bicycling. Think only about those physical activities that you did for at least 10 minutes at a time”. Regular participation is a key concept included in current public health guidelines for physical activity [26]. Therefore, both the total volume and the number of day/sessions are included in the IPAQ analysis algorithms. The IPAQ demonstrates very good levels of repeatability and fair to moderate validity when compared to accelerometer data [27].

### Knee symptom measures

Knee symptoms during the past 30 days were assessed using the Western Ontario and McMaster Universities osteoarthritis index (WOMAC) scale in CDAH Knee study. Subjects were asked about the knee pain, stiffness and physical dysfunction status during computer-assisted telephone interview. Each question was graded on a scale of 0–9, where 0 indicated no symptoms and 9 indicated the maximum intensity of the symptoms. The WOMAC is an established scale for OA research and is also validated for responsiveness of knee complaints in young population without OA [28]. Total WOMAC scores were calculated by adding the scores of 5 subscales in WOMAC knee pain, 2 subscales in WOMAC stiffness and 17 subscales in WOMAC dysfunction. Presence of any pain, stiffness and dysfunction was defined as any score ≥1. Total WOMAC knee pain was also categorised into two groups based on total WOMAC score of >5 and ≤5.

### Cholesterol measures

Venous blood samples were collected from the antecubital vein after a 12-hour fast in CDAH study approximately 5 years prior to CDAH Knee study. Serum total cholesterol and HDL cholesterol concentrations were determined enzymatically (Olympus AU5400 automated analyzer, Olympus Optical, Tokyo, Japan) and the Lipid Research Clinic procedures were followed. LDL cholesterol concentration was calculated using the Friedewald formula [29].

### Blood pressure measurements

Blood pressure was recorded while seated, after resting quietly for 5 minutes, and cuff-size (small, medium, large) was determined using arm circumference. Systolic and diastolic blood pressure measurements were taken from the right brachial artery using the Omron HEM907 digital automatic monitor (Omron Healthcare Co, Ltd, Kyoto, Japan). The mean of three readings with a 1-minute interval between each was recorded.

### Insulin resistance

Fasting plasma glucose levels were measured by the Olympus AU5400 automated analyser (Olympus, Southend-on-Sea, UK). Fasting plasma insulin was measured by a microparticle enzyme immunoassay kit (AxSYM; Abbot Laboratories, Abbot Park, IL, USA) and by electrochemiluminescence immunoassay (Elecsys Modular Analytics E170; Roche Diagnostics, Mannheim, Switzerland) with interassay standardization. Insulin sensitivity was estimated by the homeostasis model assessment (HOMA) method. HOMA is determined from fasting plasma insulin and glucose where: HOMA = fasting insulin (μU/mL) × fasting glucose (mmol/L)/22.5.

### MRI measurements

Participants had an MRI scan of their dominant knee in CDAH Knee study. MRI scans were obtained from two hospitals, which used the same type of machine (General Electric Medical Systems, Milwaukee, WI, USA). Knees were imaged in the sagittal plane on a 1.5 T whole body magnetic resonance unit with use of a commercial transmit-receive extremity coil. The following image sequence was used: (1) T1-weighted fat saturation three-dimensional spoiled gradient recall acquisition in the steady state; flip angle 55 degrees; repetition time 58 msecs; echo time 12 msec; field of view 16 cm; 60 partitions; 512 × 512 matrix; acquisition time 11 min 56 sec; one acquisition. Sagittal images were obtained at a partition thickness of 1.5 mm and an in-plane resolution of 0.31 × 0.31 (512 × 512 pixels). (2) Proton density-weighted fat saturated two-dimensional fast spin-echo coronal images at a partition thickness of 3.3 mm and an in-plane resolution of 0.31 × 0.31 (512 × 512 pixels).

BMLs were measured using the coronal proton density-weighted images and was marked as the increased signal intensity area in the subchondral bone adjacent to the osteochondral junction. BMLs were scored in medial and lateral compartment of tibia and femur using an ordinal scoring system which we used previously [11]. Subjects with no BMLs were scored as grade 0 and then the subjects with BMLs were graded according to the percentage of area of occupancy of BML in each compartment: grade 1: ≤25 % of area; grade 2: >25 % to <50 %; grade 3: >50 %.

Cartilage defects were measured using both proton density coronal images and T1-weighted sagittal images. Cartilage defects were graded in an ordinal scale as we published before [30]. Grade 0 indicated a normal cartilage, grade 1 indicated focal blistering and low (T1-weighted) or high (proton density-weighted) signal intensity area with intact surface/bottom. Grade 2 indicated a loss of thickness of less than 50 % on surface/bottom of the cartilage. Grade 3 represented a deep ulceration with loss of thickness >50 % and grade 4 indicated a full-thickness chondral wear with exposure of subchondral bone. A prevalent cartilage defect was defined as a cartilage defect score of ≥2 at any site within that compartment.

Meniscal tear was graded in medial and lateral menisci separately based on a combined Whole-Organ Magnetic Resonance Imaging Score (WORMS) scoring system [31] from grade 0 to 2 using proton density-weighted coronal and T1-weighted sagittal images. Grade 0 was a fully normal intact meniscus. Grade 1 indicated a non-displaced tear (scored as grade 1 and 2 by WORMS). Grade 2 indicated a displaced tear or maceration (scored as grade 3 and 4 by WORMS).

Meniscal extrusion was recorded on the medial and lateral menisci and was graded from grade 0 to 2 based on the proton density-weighted coronal images as published before [32]. Grade 0 was an intact meniscus without any degree of extrusion. Grade 1 indicated a partially displaced meniscus with respect to tibia and grade 2 represented a complete displaced meniscus. Meniscal lesions were defined as any meniscal tear or meniscal extrusion in the knee.

### Statistical analyses

Mean and standard deviation or the percentages of the subjects were used for calculating the characteristics of the participants. *t* test or chi-square test was used to compare the characteristics of the participants based on the presence or absence of BML. Univariable and multivariable log binomial regression was used to estimate prevalence ratio (PR) of the associations between BML (dependent variable) and knee symptoms, structural pathologies, physical activity and cholesterol (independent variable) before and after adjustment for potential confounders. If the log binomial model failed to converge, PR was estimated using a Poisson distribution and robust standard errors. Age, gender, BMI, previous knee injury and/or duration of follow-up (for cholesterol analyses only) were examined as potential confounders based on the significant associations with BMLs or our previous literature suggesting that these are important covariates. All statistical analyses were performed on IBM SPSS 19 for Mac (IBM Corp., Armonk, NY, USA).

## Results

The prevalence of BML in the knee joint was 17 % (grade 1: 10.7 %, grade 2: 4.3 %, grade 3: 1.8 %). The baseline characteristics of the participants based on the presence or absence of BMLs are presented in Table 1. Participants with BML were similar in terms of gender distribution, BMI, total physical activity and total and LDL cholesterol when compared with participants without BMLs. However, participants with BMLs had lower moderate physical activity, higher proportion with cartilage defects, meniscal lesions, and WOMAC pain, and dysfunction. HDL cholesterol was lower in participants with BMLs compared to those without BMLs.

**Table 1:** Baseline characteristics of the participants based on their bone marrow lesion status

	No BML	BML (Yes)	*P* value
	(*n* = 272)	(*n* = 55)	
Age (years)	35.2 (2.7)	36.0 (2.6)	0.053
Sex (male, %)	52	58	0.239
BMI (kg/m^2^)	25.7 (4.3)	25.6 (4.0)	0.864
Knee injury (%)	16	24	0.113
Total physical activity (min/week)	927.4 (1308.9)	932.6 (1354.1)	0.979
Total vigorous PA (min/week)	205.2 (446.6)	349.0 (987.9)	0.094
Total moderate PA (min/week)	277.3 (621.6)	122.7 (189.2)	0.001
Cartilage defects (%)	4.4	16.4	0.003
Total meniscal extrusion (%)	3.3	11.1	0.024
Total meniscal tear (%)	12	22	0.051
Total meniscal lesions (%)	13.6	27.3	0.013
WOMAC pain (yes, %)	34	45	0.084
WOMAC stiffness (yes, %)	31	41	0.075
WOMAC dysfunction (yes, %)	39	56	0.018
WOMAC pain (>5 vs ≤5, %)	11	22	0.032
HDL cholesterol (fasting, mmol/L)	1.45 (0.34)	1.34 (0.28)	0.019
LDL cholesterol (fasting, mmol/L)	2.90 (0.80)	2.87 (0.69)	0.727
Total cholesterol (fasting, mmol/L)	4.86 (0.93)	4.69 (0.74)	0.145

Two-tailed *t* tests used for differences between means; χ2 test used for proportions (percentages)

Mean (SD) except for percentages

*BML* bone marrow lesion, *BMI* body mass index, *PA* physical activity, *WOMAC* Western Ontario and McMaster Universities osteoarthritis index, *HDL* high-density lipoprotein, *LDL* low-density lipoprotein

Cross-sectional associations between demographic factors, physical activity and BMLs are shown in Table 2. Age was associated with BML in univariable and multivariable analysis. Gender, BMI, and previous knee injury were not significantly associated with BML; however, previous knee injury was associated with medial tibiofemoral BMLs (PR: 2.20, 95 % confidence interval (CI): 1.03, 4.71) in multivariable analysis. Total physical activity was not associated with BML in the knee, but moderate physical activity was protectively associated with BML before and after adjustment for age, gender, BMI and knee injury. Vigorous physical activity showed a weak but deleterious association with medial tibiofemoral BMLs (PR: 1.02, 95 % CI: 1.01, 1.03) and the association with any knee BMLs was of borderline significance. These associations remained significant after further adjustment for cartilage defects and meniscal lesions.

**Table 2:** Associations of demographic factors and physical activity with bone marrow lesions in young adults

	Univariable PR (95 % CI)	Multivariable^a^ PR (95 % CI)
Age (per year)	**1.09** **(1.00, 1.20)**	**1.10** **(1.00, 1.20)**
Sex (females vs males)	1.24 (0.76, 2.02)	1.26 (0.76, 2.08)
BMI (per kg/m^2^)	1.00 (0.94, 1.05)	0.99 (0.93, 1.05)
Knee injury (yes vs no)	1.50 (0.87, 2.60)	1.49 (0.86, 2.58)
Total physical activity (per hour/week)	1.00 (0.99, 1.01)	1.00 (0.99, 1.01)
Total vigorous PA (per hour/week)	**1.01** **(1.00, 1.02)**	1.01 (0.99, 1.02)
Total moderate PA (per hour/week)	**0.94** **(0.88, 0.99)**	**0.93** **(0.87, 0.99)**

Bold denotes statistical significance at *P* <0.05

*PR* prevalence ratio, *95 % CI* 95 % confidence interval, *BMI* body mass index, *PA* physical activity

^a^Adjusted for age, sex, BMI, and knee injury

**Fig. 1:**
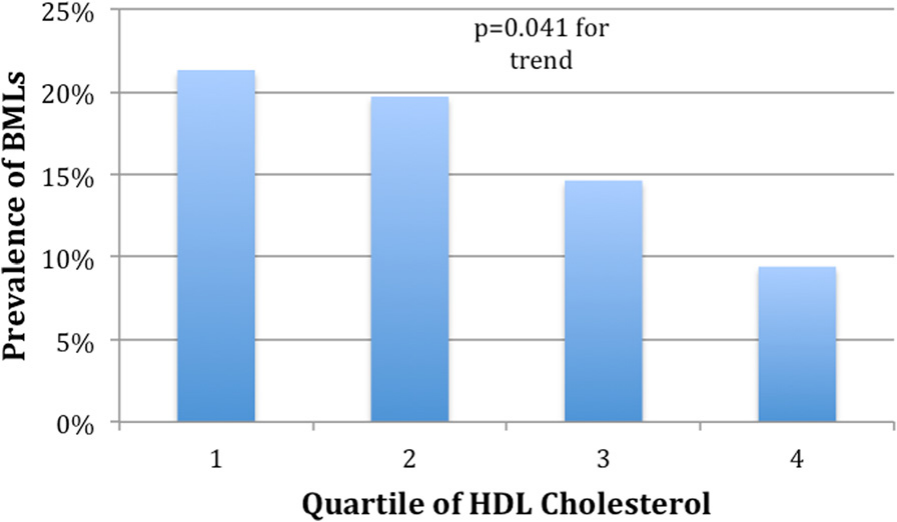
Association between prevalence of any bone marrow lesion and quartile of high-density lipoprotein cholesterol measured approximately 5 years prior. *P* value from multivariable log binomial regression after adjusting for age, sex, body mass index, knee injury and duration of follow-up. *BMLs* bone marrow lesions, *HDL* high-density lipoprotein

HDL cholesterol measured approximately 5 years prior was negatively associated with BML in univariable and multivariable analysis (Table 3). This association remained significant after further adjustment for HOMA, systolic blood pressure and waist circumference measured 5 years prior. Figure 1 shows the prevalence of BMLs based on quartiles of HDL cholesterol. Total cholesterol or LDL cholesterol was not significantly associated with BML of the knee after adjustment for age, gender, BMI, duration of follow-up and knee injury (Table 3).

**Table 3:** Association of cholesterol measured approximately 5 years prior with bone marrow lesion

	Univariable PR (95 % CI)	Multivariable^a^ PR (95 % CI)
HDL cholesterol (per mmol/L)	**0.42** **(0.20, 0.88)**	**0.36** **(0.15, 0.87)**
LDL cholesterol (per mmol/L)	0.95 (0.71, 1.27)	0.94 (0.69, 1.27)
Total cholesterol (per mmol/L)	0.83 (0.65, 1.07)	0.84 (0.66, 1.06)

Bold denotes statistical significance at *P* <0.05

*PR* prevalence ratio, *95 % CI* 95 % confidence interval, *HDL* high-density lipoprotein, *LDL* low-density lipoprotein

^a^Adjusted for age, sex, BMI, duration of follow-up and knee injury

**Fig. 2:**
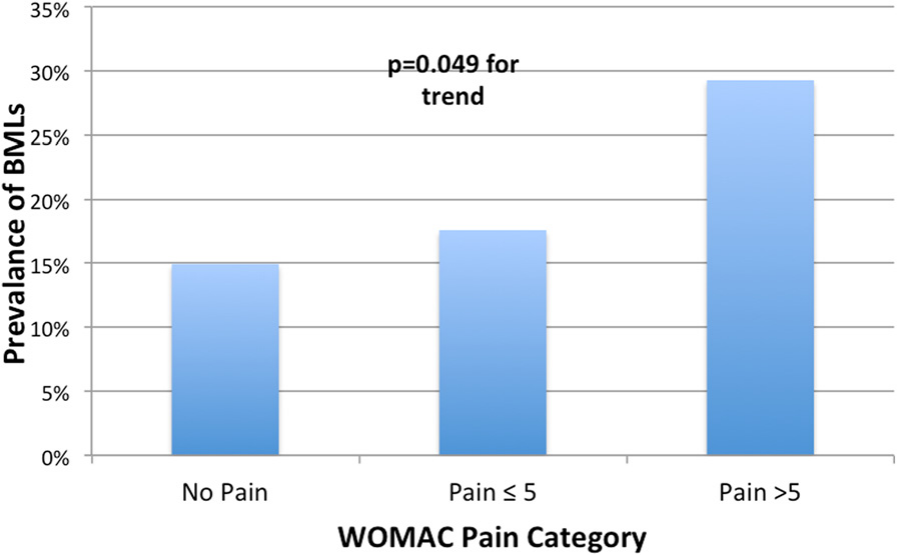
Association between prevalence of any bone marrow lesion and category of total knee pain (0–45). *P* value from multivariable log binomial regression after adjusting for age, sex, body mass index and knee injury. *BMLs* bone marrow lesions, *WOMAC* Western Ontario and McMaster Universities osteoarthritis index

Figure 2 shows the prevalence of BMLs in each pain category based on the grouping of total WOMAC pain (0, ≥1 to 5, >5). BMLs in the knee was not significantly associated with any total WOMAC knee pain (≥1 vs =0); however, when we categorised total WOMAC knee pain as >5 vs ≤5 for a severity analysis, there was a significant association with BMLs before and after adjustment for covariates (Table 4). However, the association became of borderline significance (*P* = 0.072) after further adjustment for cartilage defects, but was independent of meniscal lesions.

**Table 4:** Association of bone marrow lesions with knee symptoms and structural abnormalities

	Univariable PR (95 % CI)	Multivariable^a^ PR (95 % CI)
Symptoms		
WOMAC pain (any vs no)	1.46 (0.90, 2.35)	1.41 (0.86, 2.33)
WOMAC stiffness (any vs no)	1.49 (0.92, 2.40)	1.48 (0.90, 2.45)
WOMAC dysfunction (any vs no)	**1.75** **(1.07, 2.84)**	**1.75** **(1.07, 2.89)**
WOMAC pain (>5 vs ≤5)	**1.87** **(1.08, 3.24)**	**1.80** **(1.03, 3.14)**
Structural abnormalities		
Cartilage defects (any vs no)	**2.85** **(1.63, 4.50)**	**2.54** **(1.49, 4.34)**
Meniscal extrusion (any vs no)	**2.59** **(1.32, 5.08)**	**2.50** **(1.29, 4.82)**
Meniscal tear (any vs no)	**1.75** **(1.00, 3.05)**	1.63 (0.93, 2.86)
Meniscal lesion (any vs no)	**1.98** **(1.19, 3.32)**	**1.92** **(1.13, 3.28)**

Bold denotes statistical significance at *P* <0.05

*PR* prevalence ratio, *95 % CI* 95 % confidence interval, *WOMAC* Western Ontario and McMaster Universities osteoarthritis index

^a^Adjusted for age, sex, BMI, and knee injury

The prevalence of knee pain in grade 1, 2 and 3 BMLs were 37 %, 57 % and 67 %, respectively. The prevalence ratios of grade 1 and grade 2 or 3 BMLs (grade 0 as the reference) with WOMAC knee pain, stiffness and dysfunction are presented in Table S1 in Additional file 1. Higher grades of BMLs (grade 2 and 3 combined) were significantly associated with higher prevalence of any WOMAC pain, WOMAC pain >5 and physical dysfunction (Table S1 in Additional file 1). There was a dose–response relationship between BML grades and prevalence of WOMAC knee pain >5 (Figure S1 in Additional file 1). Presence of BML was also associated with any WOMAC dysfunction in univariable and multivariable analyses (Table 4). These associations remained largely unchanged after further adjustment for cartilage defects and meniscal lesions.

As shown in Table 4, BMLs were associated with other knee structural abnormalities including total knee cartilage defects and total meniscal extrusion, as well as total meniscal lesions defined as any tear or extrusion of the menisci. BMLs were not significantly associated with cartilage volume or bone area in these young adults (data not shown).

## Discussion

This is the first study exploring the correlates of knee BMLs in a population-based sample of young adults. Knee BMLs were surprisingly commonly being found in 17 % of participants. They were associated with increasing age, previous knee injury, increased knee symptoms and structural abnormalities such as meniscal lesions and cartilage defects. Furthermore, moderate physical activity and higher HDL cholesterol were associated with decreased while vigorous activity was weakly associated with increased BML, suggesting that BMLs in young adults are modifiable.

BMLs are commonly found in knee OA patients and are also present in non-OA populations. Prevalence of BMLs in this young population-based cohort was 17 %, which is comparable with prevalence reported previously in an older but non-symptomatic or non-OA population [33–35]. Similarly, not all participants with BMLs were symptomatic in our study. Pathology of BMLs are complex and BML zone mainly consisted of normal tissue (53 % of the area was fatty marrow, 16 % was intact trabeculae, and 2 % was blood vessels) and a smaller proportion of several abnormalities (bone marrow necrosis [11 % of area], abnormal [necrotic or remodelled] trabeculae [8 %], bone marrow fibrosis [4 %], bone marrow oedema [4 %], and bone marrow bleeding [2 %]) [36]. Subchondral bone is rich in blood supply and it is possible that the systemic and local factors can play a role in the BML pathology. We found that age was associated with increased risk of BMLs, which is consistent with a finding in a non-OA population cohort [37]. Injury was associated with medial tibiofemoral BMLs, which is in line with the finding observed in middle-aged and older adults [38]. However, BMI was not significantly associated with BMLs in this young cohort. Similar results were also observed in healthy middle-aged and older adults [37, 39]. In contrast, a systematic review has suggested a moderate level of evidence for the association between obesity measures and BMLs [40].

The current evidence of the association of physical activity with BMLs are mostly in older adults and suggests beneficial [20], detrimental [21, 22, 41] or no effect [37, 42]. Racunica et al. reported that non-vigorous activity (less vigorous activity and walking) was negatively associated with BMLs [20] in healthy older adults; in contrast, we reported that strenuous physical activity independently predicted an increase in BML size in middle-aged adults [5]. Similarly, we found that doing ≥10000 steps per day was associated with an increase in BML in older adults [22]. Asymptomatic middle-aged individuals from the “Osteoarthritis Initiative” incidence cohort had more knee BMLs in those who were more physically active, independent of confounders [21]. We found in this young population that although total physical activity was not associated with BMLs, moderate physical activity was associated with reduced BMLs in the whole knee but vigorous physical activity was weakly associated with increased BMLs in medial tibiofemoral compartment. These associations were independent of cartilage defects and meniscal lesion. These findings suggest that different levels of physical activity may have different influences on subchondral bone health in young adults.

Vascular pathology is proposed in the pathophysiology of OA. Increased popliteal artery wall thickness was associated with reduced medial tibial cartilage volume, increased rate of cartilage volume loss and a trend for BML worsening over 2 years [43]. Total cholesterol and triglycerides were associated with increased BMLs in women [19]. In our study, we found that although total cholesterol and LDL measured 5 years prior were not associated with BMLs, HDL cholesterol measured 5 years prior was significantly associated with reduced BMLs. This association was independent of other metabolic syndrome measurements such as blood pressure, waist circumference and HOMA. This is consistent with our previous longitudinal findings in older adults where HDL cholesterol was associated with BML resolution and had a protective effect on incidence of BMLs [18]. HDL cholesterol is considered to be protective against vascular pathology through cholesterol transport, anti-inflammatory, and antioxidant effects, and therefore may help to reduce BML development and progression [44].

Subchondral bone is richly innervated with nociceptive pain fibres [45]. Thus, subchondral bone could be a source of knee pain. There is increasing evidence to suggest that BMLs are associated with knee pain [4, 46–48] in older adults or knee OA patients, though some studies did not establish this association [49, 50]. A systematic review suggested that there was moderate evidence for the association between BMLs and knee pain [51]. We found that BMLs were associated with more severe knee pain when the total WOMAC pain was categorized at 5 in young adults, though this association was in part dependent of cartilage defects. Higher grades of BMLs were independently associated with greater knee pain, suggesting that BMLs in young adults would also be a source of knee pain.

Structurally, BMLs appear to be sclerotic compared with unaffected regions from the same individual based on the increased bone volume fraction and increased trabecular thickness [52, 53]. The mineral density in these lesions, however, is reduced and may render this area to be mechanically compromised, and thus susceptible to attrition [52]. Therefore, it is highly likely that BMLs can have a local mechanical effect on the knee joint. It has been reported that BMLs can predict site-specific cartilage pathology including cartilage defect progression and cartilage volume loss in older [11, 54] and middle-aged [34] adults. Similarly, BMLs are associated with meniscal pathology [55] and meniscal pathology increases the risk for the incidence and progression of BMLs in older adults [56]. This study is the first to report that in young adults, BMLs were associated with meniscal lesions and cartilage defects. We did not find any significant association with knee cartilage volume or bone area, which concurs with the results from a study in healthy middle-aged women [33], indicating that BMLs may be associated with earlier structural changes such as cartilage defects rather than later structural changes such as reduced cartilage volume in young populations.

A strength of our study was the use of a population-based sample in young adults. This study has several potential limitations. The response rate was low with only 43 % of the persons invited to participate having MRI performed. Reassuringly, there were no significant differences in age, sex, BMI, and knee injury between those with and without MRI scans, or between subjects included in this study and the remainder of the original cohort, which suggests there was no major selection bias introduced. We did not record the severity of injury in this study and therefore cannot assess the effect of injury severity on BMLs. Cholesterol measures were assessed 5 years prior and we did not have MRI measurements at that time, so cannot examine if cholesterol was associated with change in BMLs over time. We did not record separate sports activities that could have contributed to the vigorous physical activity. The strength of the association of physical activity with BML was very low and the results need to be interpreted cautiously. Most of these findings were based on cross-sectional data and therefore the causal pathways cannot be ascertained.

## Conclusions

In conclusion, BMLs in young adults are associated with knee symptoms, a history of knee injury and other knee structural lesions. Moderate physical activity and HDL cholesterol are beneficially associated with BMLs, in contrast, vigorous physical activity is weakly but positively associated with medial tibiofemoral BMLs. These suggest that BMLs are modifiable in young adults.

## Additional file

Additional file 1: Table S1. Associations between different grades of bone marrow lesions and knee symptoms. **Figure S1.** Association between different grades of bone marrow lesions and prevalence of WOMAC knee pain >5. (DOCX 25 kb)

## Abbreviations

ASHFS: Australian Schools Health and Fitness Survey
BMI: Body mass index
BMLs: Bone marrow lesions
CDAH: Childhood Determinants of Adult Health
CI: confidence interval
HDL: High-density lipoprotein
HOMA: Homeostasis model assessment
IPAQ: International Physical Activity Questionnaire
LDL: Low-density lipoprotein
MRI: Magnetic resonance imaging
OA: Osteoarthritis
PR: Prevalence ratio
WOMAC: Western Ontario and McMaster Universities Osteoarthritis Index
WORMS: Whole-Organ Magnetic Resonance Imaging Score
