# Supplementary material for: Correlates of knee bone marrow lesions in younger adults
Source: Arthritis Res Ther. 2016 Jan 26;18:31. doi: 10.1186/s13075-016-0938-9 (PMC4730612; doi:10.1186/s13075-016-0938-9)
Supplement: Additional file 1: Table S1. — Associations between different grades of bone marrow lesions and knee symptoms. Figure S1. Association between different grades of bone marrow lesions and prevalence of WOMAC knee pain >5. (DOCX 25 kb) [file 13075_2016_938_MOESM1_ESM.docx]

**Additional File 1:**

**Correlates of knee bone marrow lesions in younger adults**

^1^ Benny Antony, PhD (Benny.EathakkattuAntony@utas.edu.au)

^1^ Alison Venn, PhD (Alison.Venn@utas.edu.au)

^2^ Flavia Cicuttini, MD, PhD (Flavia.Cicuttini@monash.edu)

^3^ Lyn March, MD (Lyn.March@sydney.edu.au)

^1^ Leigh Blizzard, PhD (Leigh.Blizzard@utas.edu.au)

^4^ Terence Dwyer, MD, PhD (Terry.Dwyer@mcri.edu.au)

^5^ Andrew Halliday, MD (andrew.halliday@dhhs.tas.gov.au)

^3^ Marita Cross, PhD (maritac@med.usyd.edu.au)

^1^ Graeme Jones, MD (Graeme.Jones@utas.edu.au)

^1,2^ Changhai Ding, MD (Changhai.Ding@utas.edu.au)

^1^Menzies Institute for Medical Research, University of Tasmania, Hobart, Australia; ^2^Department of Epidemiology and Preventive Medicine, Monash University, Melbourne, Australia; ^3^Institute of Bone and Joint Research, University of Sydney, Sydney, Australia; ^4^Murdoch Childrens Research Institute, Melbourne, Australia; ^5^Department of Radiology, Royal Hobart Hospital, Australia

**Supplementary Tables:**

**Table S1**. Associations between different grades of bone marrow lesions and knee symptoms.

|  | **BMLs Grade 1***  PR (95%CI) | **BMLs Grade 2/3***  PR (95%CI) |
| --- | --- | --- |
| WOMAC pain (yes vs no) | 1.07 (0.54, 2.12) | **2.60** (**1.09, 6.17**) |
| WOMAC pain (>5 vs ≤5) | 1.24(0.50, 3.11) | **3.49** (**1.46, 8.34**) |
| WOMAC stiffness (yes vs no) | 1.41 (0.73, 2.72) | 1.81 (0.71, 4.61) |
| WOMAC dysfunction (yes vs no) | 1.49(0.77, 2.86) | **2.82** (**1.13,7.00**) |

Reference group: no BMLs.

*Adjusted for age, sex, BMI, and knee injury

PR: prevalence ratio

BMLs: bone marrow lesions

95%CI: 95% confidence interval

WOMAC: Western Ontario and McMaster Universities osteoarthritis index

Bold denotes statistical significance at p<0.05

**Supplementary Figure legends:**

**Figure S1. Association between different grades of bone marrow lesions and prevalence of WOMAC knee pain >5.**

p-value from multivariable log binomial regression after adjusting for age, sex, body mass index and knee injury

BML: bone marrow lesion

WOMAC: Western Ontario and McMaster Universities osteoarthritis index
